# Multi-Damage Detection in Composite Space Structures via Deep Learning

**DOI:** 10.3390/s23177515

**Published:** 2023-08-29

**Authors:** Federica Angeletti, Paolo Gasbarri, Massimo Panella, Antonello Rosato

**Affiliations:** 1 School of Aerospace Engineering, Sapienza University of Rome, Via Salaria 851, 00138 Rome, Italy; paolo.gasbarri@uniroma1.it; 2 Department of Information Engineering, Electronics and Telecommunications (DIET), Sapienza University of Rome, via Eudossiana 18, 00184 Rome, Italy; massimo.panella@uniroma1.it (M.P.); antonello.rosato@uniroma1.it (A.R.)

**Keywords:** structural health monitoring, deep learning, flexible structures, composite materials

## Abstract

The diagnostics of environmentally induced damages in composite structures plays a critical role for ensuring the operational safety of space platforms. Recently, spacecraft have been equipped with lightweight and very large substructures, such as antennas and solar panels, to meet the performance demands of modern payloads and scientific instruments. Due to their large surface, these components are more susceptible to impacts from orbital debris compared to other satellite locations. However, the detection of debris-induced damages still proves challenging in large structures due to minimal alterations in the spacecraft global dynamics and calls for advanced structural health monitoring solutions. To address this issue, a data-driven methodology using Long Short-Term Memory (LSTM) networks is applied here to the case of damaged solar arrays. Finite element models of the solar panels are used to reproduce damage locations, which are selected based on the most critical risk areas in the structures. The modal parameters of the healthy and damaged arrays are extracted to build the governing equations of the flexible spacecraft. Standard attitude manoeuvres are simulated to generate two datasets, one including local accelerations and the other consisting of piezoelectric voltages, both measured in specific locations of the structure. The LSTM architecture is then trained by associating each sensed time series with the corresponding damage label. The performance of the deep learning approach is assessed, and a comparison is presented between the accuracy of the two distinct sets of sensors: accelerometers and piezoelectric patches. In both cases, the framework proved effective in promptly identifying the location of damaged elements within limited measured time samples.

## 1. Introduction

As the call for more demanding aerospace technologies increasingly expands, so does the complexity and size of the structures equipped to modern satellites [1,2,3]. Successful operations in space rely on the structural integrity of these components, such as antennas and solar panels. Space structures are indeed subject to various damage risks, including thermal stresses, debris or micrometeoroid impacts, and radiation [4]. These factors, coupled with the in-orbit extreme environment, can cause progressive degradation, fatigue, and potential failures. Moreover, the challenges of structural integrity during in-orbit operative life are of course aggravated by the inherent constraints on human access, which limit applying traditional techniques to crewed vehicles. The early detection of structural damage is therefore pivotal to ensure the safety and reliability of the space mission. Any undetected damage can hinder the required performance, and even jeopardise the entire mission. On the other hand, promptly identifying structural failures can enable a proactive response and the activation of mitigation strategies to avoid the damage escalating to critical levels. Also, automated and/or autonomous failure detection systems will be a key feature of future space robotic maintenance and repair infrastructures [5,6]. 

Structural health monitoring (SHM) methods aim to assess the conditions of structural systems by integrating sensors, data acquisition assets, and advanced computational processing algorithms. SHM enables the real-time early detection of damage, and it is successfully used in aerospace vehicles [7,8,9]. In this field, some of the most commonly applied sensing technologies are piezoelectric components [10], accelerometers, and fibre-based optical sensors. Concerning space systems, several factors must be considered in the choice of sensors, among which are performance, reliability, and mass/cost ratio, especially when referring to modern lightweight structures. In this work, accelerometers and piezoelectric devices are implemented in an SHM architecture and their performance compared, as it is proven that they are more suitable to monitor larger areas of structures than fibres [11]. Moreover, they can be easily installed as plug-in components, and have limited impact on large-scale systems. 

In the past few decades, several methods have been investigated to conduct diagnosis and failure analysis for aerospace systems, mostly based on traditional methods, such as lamb wave-based approaches [12], transmissibility functions [13], and strain modes [14]. Structural quality and integrity controls are generally performed using non-destructive testing techniques for composite materials. These strategies, however, are characterised by some limitations—especially related to the need for advanced filtering, image processing solutions, and structural accessibility—which can be overcome by approaching machine learning (ML) technologies for defect classification [15]. Lately, growing interest has been shown towards the integration of ML techniques in SHM systems to enable automated damage detection, classification, and prediction. Algorithms based on artificial intelligence can analyse complex data patterns, detect subtle changes, and give real-time feedback to mission controllers in an autonomous fashion, providing good training based on stable and informative data. Among machine learning techniques, neural networks (NNs) stand out as the most effective approach for extracting information and patterns from data that are difficult to be efficiently and accurately analysed by human intervention alone. Nowadays, they have emerged as the state-of-the-art solution for various industrial and scientific applications. To cite some examples, Worden and Staszewski proposed an approach for damage location using neural networks on a composite panel for aerospace applications [16], and analysed optimal sensors distribution for impact detection on composite materials. LeClerc et al. [17] coupled a classifier and a regression algorithm in a two-step method and obtained the impact location on the scaled model of an aircraft composite wing via piezoelectric measurements. Khodaei et al. [18] developed an NN-based metamodel capable of identifying the coordinates of a damage from piezo sensor readings over a full composite stiffened panel. 

Deep learning (DL) algorithms in general and deep neural networks (DNNs) rely mainly on stacking multiple neural layers to push the boundaries of learning complex representations and patterns, at the expenses of a longer and heavier training procedure with respect to classic neural networks [19,20]. Recent advancements have explored the use of DNNs such as convolutional neural networks (CNNs) and deep recurrent neural networks (DRNNs) within the health monitoring research field. In particular, the use of the convolution operation and its associated information extraction method generally works with measured time series data by converting them into 2D graphical representations (spectrograms) [21] or by extracting damage-sensitive features from the measured vibration response [22,23]. However, as the collected data usually consist of sequential time series signals, the most efficient deep learning architecture to be applied for prediction and classification falls within the RNNs domain [24]. In particular, it has been demonstrated that the advanced sub-class named Long Short-Term Memory (LSTM) networks [25,26] yields superior results when analysing and classifying time series without the need for complex preprocessing and hand-crafted feature extraction [27].

This paper introduces an LSTM architecture to investigate structural health monitoring for space systems, given its relatively new application to this sector. Previous implementations have been limited to very simple structures [28] or focused on a broader concept of damage and/or anomaly detection, such as satellite image time series [29] or telemetry signal [30] defects. In this scenario, the authors developed a methodology to investigate the presence of structural damage on the flexible appendages of satellites using data collected from distributed sensors during standard in-orbit operations such as attitude manoeuvres. This approach contributes to the maturing of space technologies both for the active monitoring of their health status and for the enhancement of traditional methods of damage identification. The main novelties presented in this work are further discussed in the Section 1.1. 

The paper Is divided as follows: firstly, the spacecraft dynamics is presented in Section 2, including a description of the flexible spacecraft and the mathematical formulation implemented in the simulator of attitude manoeuvres. Then, Section 3 presents the damage scenarios, introducing the considered structural damage as space debris hits and the number and location of sensors. In Section 4, the deep learning architecture for damage classification is described, while a description of the training dataset generation is also provided. Section 5 presents the main results and shows the performance of the trained classification network. The relevant findings are discussed in Section 6, while Section 7 presents the conclusions, along with prospective future research areas.

### 1.1. Highlights of the Paper’s Contributions

The current work builds on the approach developed by the authors [31,32], with the aim to further structure the method, not only by testing the DL framework on a new case study, but also challenging its classification capabilities. Indeed, based on the knowledge and experience gained in previous work, the present study aims to introduce a general and detailed end-to-end architecture and guidelines to be followed for performing the SHM of space structured based on LSTM-NNs. The main innovative elements are as follows:

- The methodology is applied to a new challenging study case in terms of the impact of space debris on the global spacecraft dynamics (and, in detail, on solar panels instead of antenna truss structures). This implies that not only different dynamics, but also different structural elements are considered in this study.
- The performance of the DL architecture is assessed by comparing the signals generated by two sensing networks: one based on accelerometer sensors and the other one on distributed piezoelectric patches.
- A procedure is presented to identify a subset of high-risk candidate failures to be detected by the SHM system based on modal strain energy (MSE) to build the dataset for the training.
- A more complex application in terms of higher dimensionality of the multi-class identification problem (i.e., classification of more than two damages at the same time) is investigated, obtaining information not only about the presence, but also the location of the damage.
- The structure and damage entity are implemented considering the equivalent properties and effects on a traditional composite aerospace structure, in particular, an aluminium honeycomb, by using information available in literature concerning the experimental debris impact on such structures.

## 2. Spacecraft Dynamics

To assess the effectiveness of the damage classification architecture, we examine the case of a spacecraft equipped with symmetric solar panels. This satellite model, which serves as a representative example of a realistic Earth Observation (EO) satellite, comprises a parallelepiped central platform and two solar arrays measuring 1 × 3 m each. The arrays are composed of two subpanels of 1 × 1 m. 

**Figure 1:**
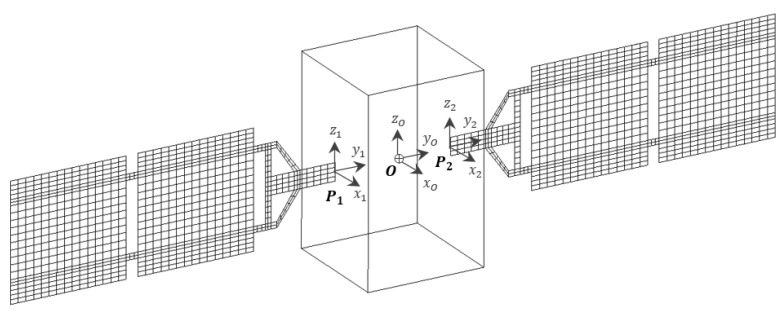
Spacecraft model.

Specifically, the panels are connected to the central platform, which is considered rigid compared to the flexible appendages. The attachment points $P_{1}$ and $P_{2}$ are defined in the spacecraft’s reference frame, whose origin O is located at the centre of gravity of the vehicle (please see Figure 1). Each array is supported by a structure made of aluminium honeycomb, while an aluminium yoke is designed to replicate the attachment of the panel to the platform. Since the central hub is assumed to be rigid, each flexible substructure is directly assembled in MSC Nastran, using rigid body element connections to link them to a single node coinciding with the point O. The relevant data including the inertial properties, modal participation factors, and natural frequencies of the flexible structure, computed with respect to O, are imported into a MATLAB^®^ R2021a environment to implement the dynamics of the flexible spacecraft as described in Section 2.2. The inertial properties of the satellite bus are presented in Table 1.

**Figure 2:**
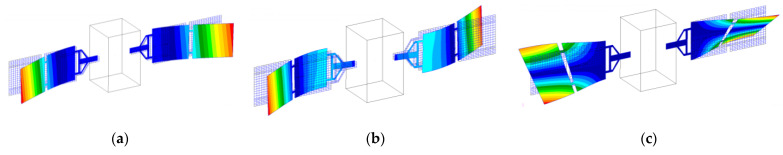
Spacecraft free-free modes: (**a**) first mode at 0.97 Hz, symmetric bending; (**b**) second mode at 1.58 Hz, antisymmetric bending; (**c**) third mode at 3.27 Hz, torsion.

Traditionally, a panel-like structure shows three different constrained modes, corresponding to bending and torsion with respect to the main coordinated axes. When mounted on a satellite hosting multiple flexible appendages, the flexible modes of one structure interact with the structural dynamics of the others. The set of modes of the assembled spacecraft is illustrated in Figure 2, along with an overview of the complete system. The resulting modal behaviour is characterised by both symmetric and antisymmetric bending modes, which are excited by translation and attitude manoeuvres, respectively.

### 2.1. Composite Equivalent Material

To meet the size and weight limitations during launch, solar panels need to be lightweight, yet strong and stiff. Hence, an aluminium honeycomb composite material is chosen here to design each subpanel of the array supporting structure. However, the use of a composite material calls for careful consideration of its structural stiffness properties. Typically, a three-layer composite model is employed in a finite element environment to replicate such material behaviour [33]. Consequently, each layer is individually modelled, potentially requiring a large number of elements. Nevertheless, it is feasible to develop an equivalent stiffness model of the composite plate using a reduced number of elements [34,35]. 

**Figure 3:**
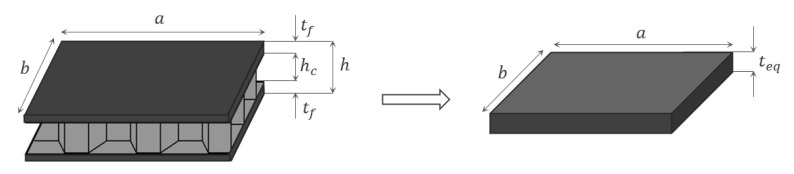
Equivalent plate theory illustration for honeycomb plate.

To decrease the complexity of the spacecraft finite element model, an equivalent representation of the multi-layer composite structure is considered as a single-layer panel, as illustrated in Figure 3. 

Traditionally, the material used for the solar panel honeycomb composite structure is Aluminium Alloy 5052 for both faces and core. The corresponding geometrical and mechanical properties are listed in Table 2.

The equivalent thickness $t_{eq}$, and stiffness moduli $E_{eq}$ and $G_{eq}$ of the one-layer equivalent model are obtained by solving the equations in the available literature for an aluminium honeycomb panel [35], as follows:

(1) $$t_{eq}=\sqrt{3h_{c}^{2}+6h_{c}t_{f}+4t_{f}^{2}},\;E_{eq}=\left(2t_{f}E_{f}\right)/t_{eq},\;G_{eq}=\left(2t_{f}G_{f}\right)/t_{eq}$$

The equivalent data for the 10 mm sandwich panel in Table 2 are $t_{eq}=0.0156\;m$, $E_{eq}=90\;\mathrm{GPa}$, and $G_{eq}=3.31\;\mathrm{GPa}$. Also, the plate equivalent density can be straightforwardly computed to maintain the same mass of the initial sandwich, based on the equivalent thickness of the one-layer structure. 

### 2.2. Governing Equations

This section presents the mathematical formulation describing the dynamics of a flexible spacecraft in a gravitational field. Since piezoelectric sensors will be considered as potential SHM devices in Section 3, they are included in the following equations.

By following a Lagrangian approach [36], it becomes possible to obtain the nonlinear dynamics theory to simulate translational and rotational manoeuvres for a flexible satellite. For the sake of brevity, only the final equations of motion are provided in this paper, as the detailed steps leading to this formulation are already found in previous literature [37,38]. Generally, the system state vector can be defined as follows:

(2) $$X=\left[X_{O},\theta ,\eta \right]$$

where $X_{O}$ represents the position of the platform’s centre of gravity (*O*) relative to an ECI inertial frame, θ is the attitude of the body reference frame in relation to the inertial system, and η denotes the modal amplitudes of a flexible appendage attached to a central satellite platform.

Therefore, the full nonlinear governing equations of the system can be written as reported in Equation (3) as

(3) $$M_{t}^{O}\ddot{X}+C_{t}\dot{X}+K_{t}X+N_{nl}=F_{t}^{O}$$

where $M_{t}^{O}$ is the total mass matrix of the system, $C_{t}$ and *K_t_* are the damping and stiffness matrices, $N_{nl}$ contains the nonlinear dynamics terms, and $F_{t}^{O}$ the generalised forces (forces, torques, and projection of forces on the modal base). In particular, the total mass matrix can be written as

(4) $$M_{t}^{O}=\left[\begin{array}{ccc} \left(\sum_{i}^{N}M_{A_{i}}\right)+M_{b} & \sum_{i}^{N}{\tilde{p}}_{O}^{A_{i}\times } & \begin{array}{ccc} L_{k}^{A_{1}} & \cdots & L_{k}^{A_{N}} \end{array}\\ \sum_{i}^{N}{\tilde{p}}_{O}^{A_{i}\times }{}^{T} & J_{O}^{A_{i}} & \begin{array}{ccc} S_{k}^{A_{1}} & \cdots & S_{k}^{A_{N}} \end{array}\\ \begin{array}{c} L_{k}^{A_{1}{}^{T}}\\ \vdots \\ L_{k}^{A_{N}{}^{T}} \end{array} & \begin{array}{c} S_{k}^{A_{1}{}^{T}}\\ \vdots \\ S_{k}^{A_{N}{}^{T}} \end{array} & \begin{array}{c} \begin{array}{ccc} I_{1} & \cdots & 0 \end{array}\\ \begin{array}{ccc} 0 & \ddots & 0 \end{array}\\ \begin{array}{ccc} 0 & \cdots & I_{N} \end{array} \end{array} \end{array}\right]$$

with $M_{A_{i}}$ being mass matrices of the *i*-th appendages, with i=1, ..., N, $M_{b}$ mass of the satellite platform, ${\tilde{p}}_{O}^{A_{i}\times }$ is the skew matrix containing the static moment of the system, with respect to the spacecraft centre of gravity *O* in the body reference frame, $J_{O}^{A_{i}}$ is the total moment of inertia of the system with respect to *O* in the body frame, $L_{k}^{A_{i}}$ and $S_{k}^{A_{i}}$ include the translation and rotation modal participation factors (coupling with the rigid motion) respectively, while $I_{k}$ is the identity matrix (modes are normalised with respect to mass). Moreover, the matrices ***C***_*t*_ and $K_{t}$ are defined as

(5) $$C_{t}=\left[\begin{array}{ccc} 0 & 0 & 0\\ 0 & 0 & 0\\ 0 & 0 & 2\Sigma \Omega \end{array}\right],\;\;\;\;\;\;K_{t}=\left[\begin{array}{ccc} 0 & 0 & 0\\ 0 & 0 & 0\\ 0 & 0 & \Omega^{2} \end{array}\right]$$

with Ω being diagonal matrix containing all frequencies of the *N* appendages cantilevered to the satellite, and Σ being the diagonal matrix including the *k*-th damping factor of the corresponding elastic mode. The term $F_{t}^{O}$ reads as

(6) $$F_{t}^{O}=\left[f_{O},\;\tau_{O},{\tilde{f}}_{O}\right]$$

where $f_{O}$ and $\tau_{O}$ are the forces and torques applied at the centre of gravity, respectively, while ${\tilde{f}}_{O}$ are the forces projected on the modal base. The extended expression of the terms $N_{nl}$ can be found in [37] and will not be presented here, as they are not the focus of the current research. In this work, the number of appendages *N* is equal to 2 (two solar arrays).

#### 2.2.1. Piezoelectric Formulation

In case of piezoelectric materials implemented on the passive structure, the presented equations must be coupled with an additional sensing equation. Piezoelectric properties are generally described by a set of constitutive equations [39], as follows:

(7) $$S_{ij}=s_{ijkl}^{E}T_{kl}+d_{kij}E_{k}\;D_{i}=d_{ikl}T_{kl}+\varepsilon_{ik}^{T}E_{k}$$

where $S_{ij}$ is the field strain, $T_{kl}$ indicates the stress field, $E_{k}$ the electric field, and $D_{i}$ the electric displacement. Also, $d_{kij}$ is the piezoelectric strain coefficient, $s_{ijkl}^{E}$ the mechanical compliance at constant electric field, and $\varepsilon_{ik}^{T}$ the dielectric permittivity at zero mechanical stress. The sensor device can be modelled by using finite elements, as already achieved with the passive structure. Indeed, a piezo patch consists of a single layer of piezoelectric material between two electrodes, which can produce an electric charge when subjected to structural deformation. The charge $Q_{s}$ is collected on the electrodes of the piezoelectric sensor and processed by a current amplifier. When the electrodes of a piezoelectric sensor are connected to an operational amplifier, they can be regarded as short-circuited and the electric field through the piezo can be set to zero. Therefore, the voltage collected on the sensor faces will be:

(8) $$\varphi_{s}=-Q_{s}/C_{f}$$

where $C_{f}\;$represents the feedback capacitance of the charge amplifier. The complete derivation of a finite element-modelled patch can be found in [38,39,40], while only the final sensing equation is introduced here. Under the assumption of a Euler–Bernoulli beam, and a sensor with constant width, the charge $Q_{s}$ can be derived as

(9) $$Q_{s}=-z_{m}d_{31}b_{p}\left(w^{\prime }\left(b\right)-w^{\prime }\left(a\right)\right)$$

where $z_{m}$ is the patch distance from the passive structure neutral plane, $d_{31}$ is the piezo electromechanical coefficient, $b_{p}$ is the sensor width, and $w^{\prime }$ is the integral of the structural curvature. It can be noticed that the sensor output is proportional to the difference of the slopes (i.e., rotations) at the extremities of the sensor strip. The rotations are reconstructed via the finite elements structural model illustrated in Figure 1. 

It should be noticed that, generally, the mass and stiffness of the piezoelectric patches should be added to the passive structure. However, the sensor properties can be neglected when mounted on large structures due to their very limited mass, dimensions, and impact on the structure modal dynamics (a sensor model patch P-876 from PI is considered with dimensions of 61 × 35 × 0.4 mm, and a mass below 5 g [41]), and are therefore not considered in this study. 

## 3. Damage Scenario

In the following paragraphs, the simulated structural damages are introduced, and those areas where failures may cause critical damage are discussed. 

### 3.1. Damages on Solar Panels

**Figure 4:**
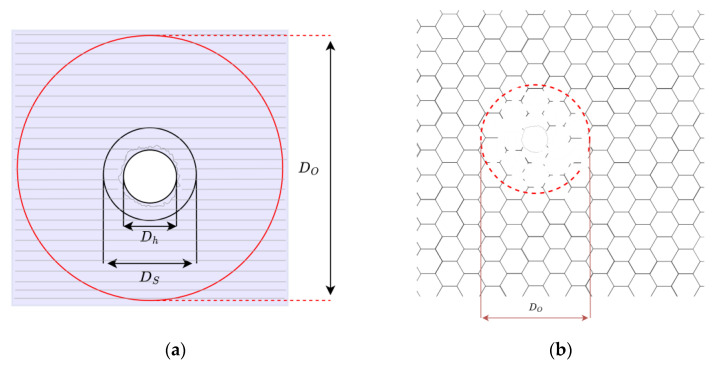
Features of samples after impact. (**a**) Representation of damage zones: $D_{h}$ perforation area, $D_{S}$ fracture zone, $D_{O}$ extended fracture zone. The diameter of the damaged honeycomb portion is assumed in the order of magnitude of the $D_{O}$ zone, in agreement with data in [42]; (**b**) impact view from the back of the honeycomb panel.

In this research, damages are considered as resulting from space debris hits, causing a perforation in the solar cells and the destruction of a wider area of the honeycomb substrate (please refer to Figure 4). The dimension of damage is assumed as not exceeding an area of 5 × 5 cm^2^, which is a representative size for high-velocity impacts for aluminium honeycomb, as demonstrated by Kunbo et al. [42]. Hence, the damage can be assumed to be a complete failure of one or more shell elements. To mathematically replicate this behaviour, those finite elements corresponding to the damaged area of the structure are considered to not contribute to the stiffness of the final structural model created in the MSC Nastran suite. In this work, damage is only simulated on one symmetric solar panel for representative purposes.

**Figure 5:**
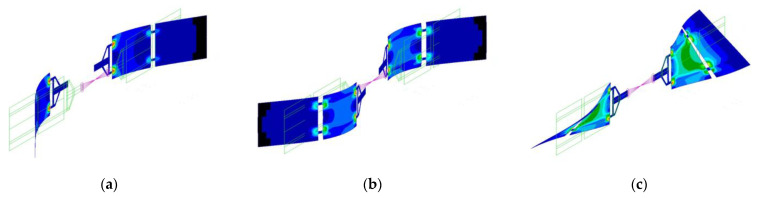
Modal strain energy (MSE) map coloured from red (areas with higher MSE density) to blue (lowest MSE density): (**a**) first mode; (**b**) second mode; (**c**) third mode.

**Figure 6:**
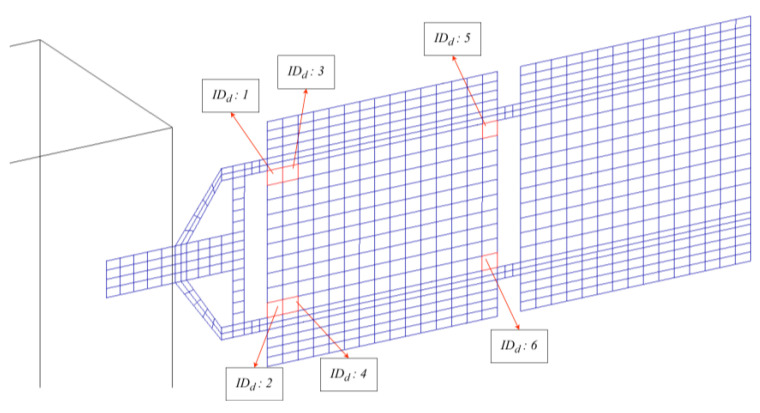
Damage configuration.

At the same time, the modal strain energy (MSE)—defined as the amount of elastic energy stored in a finite element—associated to the flexible appendages is computed using the “healthy” (i.e., with no damage) finite element model of the spacecraft. The related MSE map is used to identify the locations of the elements whose change in mechanical properties could be more problematic for the global dynamics of the system. The objective is to avoid building a heavy set of data including damage all over the structure (also damage associated with low risk, i.e., inducing a negligible change in the modal properties of the satellite), potentially leading to an excessively high-dimension multivariate classification problem. Instead, the approach proposed here is to discriminate a set of potential critical damages, to be identified via the deep learning architecture, based on MSE concentration. The elements to build the dataset are retained based on a threshold on the MSE density value. The MSE concentration is depicted in Figure 5. In detail, the areas with higher values are coloured in red (indeed, as expected for bending and torsional modes, high-MSE regions can be observed in correspondence with the attachment point of the first subpanel to the yoke), while the zones with medium and low MSE are highlighted in orange and green colours, respectively (those areas can be noticed at the attachment point of the two subpanels for the first two bending modes, and in the central area of the first subpanel for the third mode). Only elements contained in areas from red to green colours in Figure 5, which are at the same time common to all three modes, are considered in the dataset. Each point of damage will correspond to a damaged structural model used to build up the training database, as further described in Section 4.2. The faulty elements and corresponding IDs are presented in Table 3, while the damage distribution is depicted in Figure 6.

### 3.2. Sensing Networks

A diverse range of sensing technologies can be utilised to detect damage onboard satellites. These include cameras for image processing and distributed devices such as accelerometers and piezoelectric materials. Vision-based approaches to damage identification can benefit from the fact that cameras are sometimes installed to monitor other system functionalities, such as the deployment of flexible appendages, as occurred—under lucky circumstances—onboard Sentinel-1A [4]. Although cameras, if available, can prove effective compared to other sensors, they are highly sensitive to lighting conditions, exposition, noisy background, and field of view. As this paper aims to identify local failures with a versatile and robust approach applicable in different conditions, the focus is addressed towards the implementation of a set of distributed sensors. In particular, accelerometers [43] and piezoelectric devices [44] are shortlisted as promising candidates to be used in the space environment, as they are already space-qualified, add limited mass and costs, and can be straightforwardly mounted to the system. 

**Figure 7:**
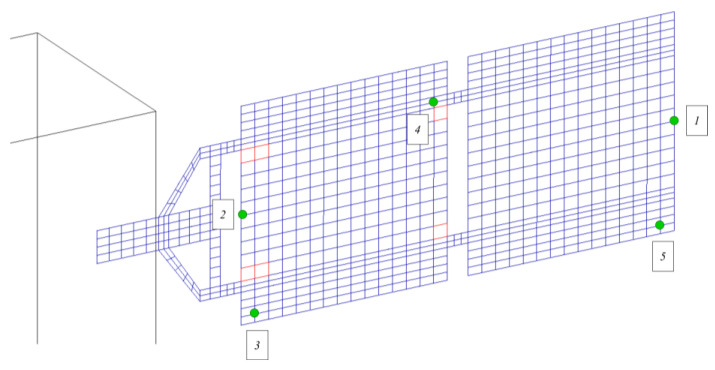
Position of accelerometers.

The sensing networks implemented, tested, and compared here are based on accelerometers and piezoelectric patches. In this section, the placement of the two different types of sensors on the structure is discussed. As the system is symmetric, a set of three-axis accelerometer sensors is installed only on one solar array for validation purposes. The same configuration can be mirrored on the other panel for a complete SHM architecture. In particular, the position of the sensors (indicated with labels from “1” to “5”) is depicted in Figure 7. The devices are placed in positions where the system dynamics response in terms of displacements is higher (i.e., the tip of the array), and in the vicinity of the most critical failure areas. 

**Figure 8:**
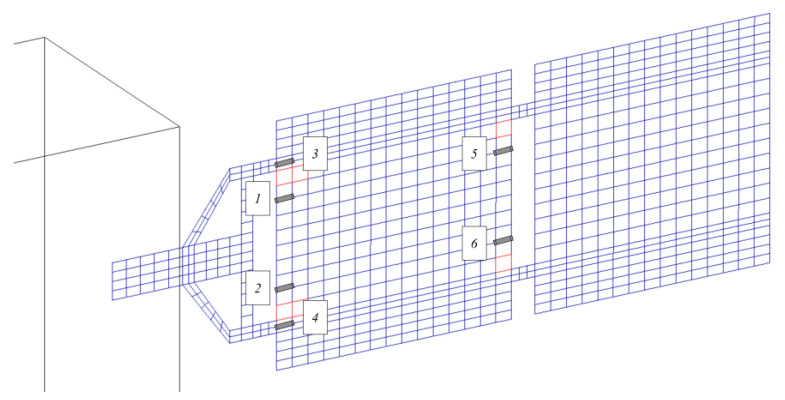
Position of piezoelectric patches.

The positioning logic of piezoelectric sensors is based on the algorithm developed and tested in the authors’ previous work [45]. In detail, piezo sensors are strategically placed in proximity to those locations experiencing higher structural deformations, corresponding to areas with the highest MSE. A total of six piezoelectric patches, marked with labels from “1” to “6” in Figure 8, were placed on the panel. 

It should be noted that the number of sensors to be implemented can be another parameter to be optimised depending on the specific application. This results in a trade-off between system complexity and desired damage identification accuracy. More details on the behaviour of the network with respect to the number of sensors is provided in Section 5.

## 4. Deep Learning Network

Deep recurrent neural networks have recently become the standard for time series classification issues. Indeed, learning models that can link information from the distant past to the current samples are increasingly being adopted in those real-world use cases that concentrate around sequences with many observations. This section provides details on the deep learning architecture used to identify the structural damage, and discusses how such a network is trained. Since the spacecraft is generally designed to carry out a pre-defined set of manoeuvres during its operative life, the data collected during such motions can be further used to investigate the health status of the system. By analysing such information, the DL network is trained to efficiently discern whether there is damage to the panel. 

**Figure 9:**
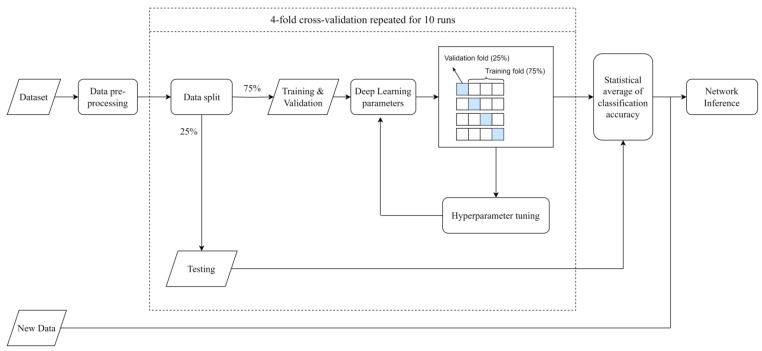
Flowchart of the adopted deep learning approach.

In this work, the structural damage identification problem is addressed as a classification task, where the time series provided by a network of sensors (DNN input) are associated with a specific label (DNN output), one for each simulated damage scenario. In detail, we use a deep bi-directional Long Short-Term Memory (Bi-LSTM) network, particularly useful when classifying lengthy sequences for a wide range of problems [46]. A flowchart of the deep learning approach is presented in Figure 9. More details on the network architecture, complexity, and training process are provided in Section 4.1, while insights on the dataset generation and data pre-processing are discussed in Section 4.2. 

**Figure 10:**
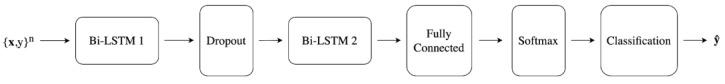
Deep learning model: {x,y} are the input/output pairs (i.e., the training set), and $\hat{y}$ is the predicted output.

The overall DNN architecture, depicted in Figure 10, consists of an input layer, two stacked Bi-LSTM layers, a dropout layer, a dense layer, a Softmax layer (which is used to predict the probability of a damage scenario), and a final classification layer (that transforms the predicted probability distribution in labels). The output of the model is a binary vector $\hat{y}$ of dimension *n* containing the prediction results. The network model suggested in this article was chosen as the most straightforward stack that could be used with this setup.

### 4.1. Network Architecture

**Figure 11:**
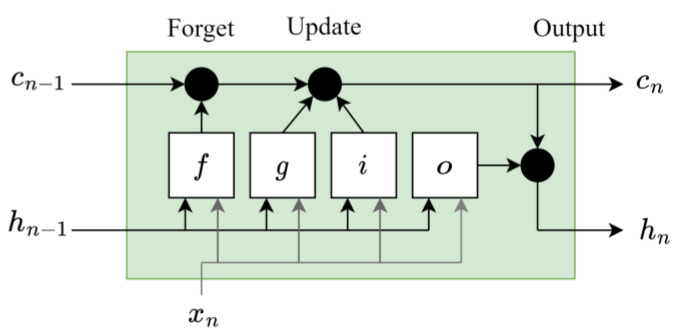
LSTM hidden unit structure.

Generally, each LSTM layer consists of $N_{h}$ recurrently connected hidden units, which compute, at the time n, the scalar output $h_{n}^{m}$ (i.e., the “hidden state”) and the scalar “cell state” $c_{n}^{m}$, where $m=1,\ldots ,N_{h}$. The whole hidden state and cell state of the LSTM layer is then defined by the column vectors $h_{n}\in R^{N_{h}}$, which include the related scalar values of the hidden units. Each unit (represented in Figure 11) takes in input the $x_{n}\in R^{N_{i}}$ (vector including the current and $N_{i-1}$ previous time samples), the previous hidden states $h_{n-1}$ and the cell states $c_{n-1}$ (coming from the unit itself and from the other units), and then it computes its hidden and cell states recursively.

At each time step, information is added to or removed from the cell state via the “gates” in each unit. Each gate output is computed at time n in the *m*-*th* hidden unit as follows:

- Input gate (indicated with the letter *i* in Figure 11) defines how much of the current input is let through for computing the new state as
  (10) $$i_{n}^{\left(m\right)}=\sigma_{g}\left(w_{i}^{\left(m\right)}x_{n}+r_{i}^{\left(m\right)}h_{n-1}+b_{i}^{\left(m\right)}\right)$$
  where $\sigma_{g}\left(\cdot \right)={\left(1-e^{-\left(\cdot \right)}\right)}^{-1}$ is a sigmoid activation function.
- Forget gate (marked with the letter *f* in Figure 11) establishes how much of the previous state pass through as
  (11) $$f_{n}^{\left(m\right)}=\sigma_{g}\left(w_{f}^{\left(m\right)}x_{n}+r_{f}^{\left(m\right)}h_{n-1}+b_{f}^{\left(m\right)}\right)$$
- Cell candidate (indicated with the letter *g* in Figure 11) selects the memory of the past as
  (12) $$g_{n}^{\left(m\right)}=\sigma_{c}\left(w_{g}^{\left(m\right)}x_{n}+r_{g}^{\left(m\right)}h_{n-1}+b_{g}^{\left(m\right)}\right)$$
  where $\sigma_{c}\left(\cdot \right)=\tan h\left(\cdot \right)$ is a hyperbolic tangent activation function.
- Output gate (mentioned with the letter *o* in Figure 11) regulates how much of the internal state will be exposed to the external network (higher layers and successive time steps) as
  (13) $$o_{n}^{\left(m\right)}=\sigma_{g}\left(w_{o}^{\left(m\right)}x_{n}+r_{o}^{\left(m\right)}h_{n-1}+b_{o}^{\left(m\right)}\right)$$

The vectors $w_{j}^{\left(m\right)}\in R^{N_{i}}$ are the LSTM input weights of the gates, while the $r_{j}^{\left(m\right)}\in R^{N_{h}}$ the LSTM recurrent weights and $b_{j}^{\left(m\right)}\in R$ the scalar biases (with j=[i,f,g,o] index of the specific gate). Hence, the cell state of the unit can be written as

(14) $$c_{n}^{\left(m\right)}=f_{n}^{\left(m\right)}c_{n-1}^{\left(m\right)}+i_{n}^{\left(m\right)}g_{n}^{\left(m\right)}$$

and the hidden state reads as follows:

(15) $$h_{n}^{\left(m\right)}=o_{n}^{\left(m\right)}\sigma_{c}\left(c_{n}^{\left(m\right)}\right)$$

The final output ${\hat{y}}_{n}\in R^{N_{o}}$ of the LSTM network at the time *n* is computed by the fully connected layer (see also Figure 10) as

(16) $${\hat{y}}_{n}=W_{d}h_{n}+b_{d}$$

where $W_{d}\in R^{N_{o}\times N_{h}}$ are the weights of the layer, and $b_{d}\in R^{N_{o}}$ the biases.

**Figure 12:**
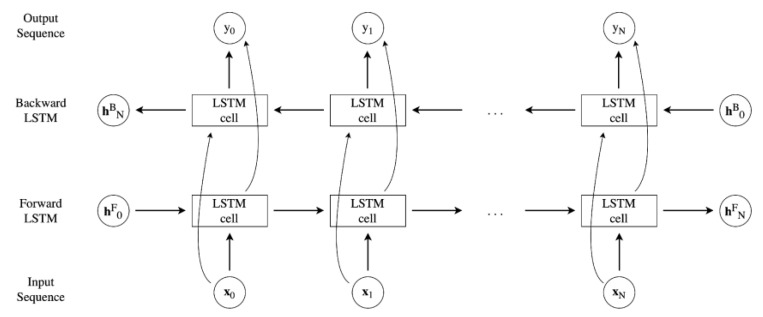
Bi-LSTM unrolled structure.

Whereas conventional unidirectional LSTMs retain solely the historical sequence information, Bi-LSTMs process each training sequence in both forward and backward directions through two distinct recurrent networks, both linked to the same output layer. This configuration captures insights from both past and future contexts, thereby enhancing the capacity for causal classification of information [47]. The Bi-LSTM applies a first LSTM on the input sequence in the prescribed order, and then flips the sequence and feeds the second LSTM, in contrast to the traditional, unidirectional LSTM model. It has been demonstrated [48] that the application examined in this study, multivariate time series analysis and classification, benefits more from the Bi-LSTM architecture, which is trained in both directions. Figure 12 reports a thorough diagram of the Bi-LSTM model’s operation. The hidden state $h_{j}^{i}$ refers to the *j*-th time step of the *i*-th (backward or forward) LSTM, and the input size is equal to *N*.

The network’s correct input sequence is created by the sequence input layer. The two Bi-LSTM layers are responsible for determining the temporal relationships between the data, and two stacked layers are recommended over one layer to increase the model’s predictive power. To prevent overfitting, a dropout layer is added in between the Bi-LSTM layers. To translate the output of the Bi-LSTM layer to the required output size, a fully connected (FC) layer is layered on top of the final Bi-LSTM unit. To allow the final classification layer to be able to conduct binary classification, the Softmax layer converts the output of the FC layer into probability values that total to one (see Figure 10).

In the DBLSTM scheme, the number of Bi-LSTM hidden units is denoted as $N_{h}^{1}$ in the first layer and $N_{h}^{2}$ in the second layer. These are blocks (that is, computational units) that are repeatedly connected, and their number should be optimised based on the specific application and data. 

#### Network Complexity

Regarding the complexity of the proposed approach, we should distinguish as usual between the training and the inference phase. The latter strongly depends on the custom hardware, possibly used onboard as in [49,50], which will perform the algebraic operations involved in each layer of the proposed architecture. As they are basically associated with matrix-vector multiplications, even in the recurrent Bi-LSTM layers, the inference time will scale up according to the computational power of the adopted hardware (such as GPU, DSP, and custom FPGA) and the related energy constraints. In spite of these considerations, the inference time is usually in the order of some microseconds per input [50,51].

Regarding the training phase, usually performed off-line on a general-purpose server or workstation, the main complexity is related to cross-validation and hyperparameter tuning, thus repeating the core training algorithm many times for the estimation of the adopted DNN’s parameters, as represented in Figure 9. The training algorithm used in this paper is the Adaptive Moment Estimation (ADAM), with an initial learning rate set to 0.08 to train all models. In addition, after every 20 epochs, the learning rate is reduced by a factor of 0.5. The methodology used for the DNN training and testing is the so-called “key-folding”; in particular, a four-fold structure has been chosen. Input/output pairs are selected in a random manner to be part of either the train or the test batches during the procedure, which is repeated 10 times to ensure statistical significance (as illustrated in Figure 9). However, the algorithm is designed to ensure that the sets used for network testing in each iteration do not include any pairs that were previously chosen for testing purposes. The statistical average of the classification accuracy on the test set, considering various folds and runs, was utilised to assess the ultimate performance of the model. The resulting performance metrics are presented in the tables in Section 5.1 and Section 5.2. 

Moreover, it is necessary to optimise specific hyperparameters of the overall architecture. In detail, the best combination of number of units for the first and the second Bi-LSTM was heuristically searched in the range from 1 to 100, with steps of five units. The other investigated hyperparameters were a dropout rate of 0.1, a maximum of 100 epochs (affecting the mini-batch size), a regularisation factor of 0.001, a gradient decay factor of 0.9, and validation patience of 40 epochs (for accelerometer-based network) and 60 epochs (for piezoelectric sensors) before early stopping. All of the experiments were performed on a machine using MATLAB^®^ R2021a, with a i7-10875H processor (eight cores at 2.30 GHz), and an NVIDIA GeForce RTX 2070 (2304 cores at 1.4 GHz), with 16 GB of GDDR5 RAM. The total network training time was equal to 0.2 GPU hours for the bi-classification problems in Section 5.1, and 0.6 GPU hours for the multi-classification application described in Section 5.2. 

The complexity of the network in terms of learnable parameters is also presented in Table 4. The analysed architecture pertains to the problem of a five-class damage identification, analysed in Section 5.2. The network is composed of 20 hidden units for the first Bi-LSTM layer, 10 hidden units for the second Bi-LSTM layer, and five neurons for the fully connected layer. As two different types (and numbers) of sensors are compared in this work, namely, accelerometers and piezoelectric devices, the network input size will vary accordingly, as reported in Table 4. The learnable parameters for both weights (input and recurrent for the LSTM layers) and biases are presented in a matrix/vector shape, as follows:

- Bi-LSTM Input weights: $\left[8\;N_{h}\times N_{in}\right]$, with $N_{h}$ number of hidden units and $N_{in}$ number of input features.
- Bi-LSTM Recurrent weights: $\left[8\;N_{h}\times N_{h}\right]$.
- Bi-LSTM Biases: $\left[8\;N_{h}\times 1\right]$.
- Fully connected layer weights: $\left[\;N_{out}\times N_{inp}\right]$, with $N_{out}$ and $N_{inp}$ number of outputs and inputs, respectively.
- Fully connected layer biases: $\left[\;N_{out}\times 1\right]$.

It should be noted that, in the proposed architecture, the number of outputs $N_{out}$ is generally equal to the classes of the damage identification problem. In Table 4, the number is five as the architecture for the most challenging experiment of Section 5.2 is presented. However, concerning the two bi-classification applications in Section 5.1, the network architecture is the same, but with different output numbers $N_{out}=2$.

### 4.2. Training Set Generation

Given that the classification problem herein studied can be formulated as a supervised one, the proposed solution based on DL is indeed data-driven; its performance and accuracy for real-world applications highly rely on the quality and quantity of the available data. Therefore, after designing the undamaged model of the solar arrays, and setting up both the damaged structural sub-models and the location of the sensors, the training dataset can be created using a high-fidelity simulator based on the mathematical formulation in Section 2.2. The overall process is a robust end-to-end approach to perform SHM for orbiting satellites, and can be described as follows:

- Relevant structural information, such as frequencies, modes, and modal participation factors, are extracted from $N_{d}=7$ structural models, each corresponding to one of the scenarios listed in Table 3. Such data are used to set up the nonlinear flexible satellite simulator to carry out a pre-defined set of $N_{m}=231$ different attitude manoeuvres. For each motion, the sensing networks record and produce measurements as time histories of accelerations (if accelerometers) or voltages (if piezoelectric devices). A quaternion-based proportional–derivative control law is applied to exert the target control torque $\tau_{O}$ to the spacecraft:
  (17) $$\tau_{O}=-K_{p}q_{e}sign\left(q_{0}\right)-K_{d}\omega$$
  where $K_{p}$ and $K_{d}$ are the proportional and derivative gains matrices, respectively, $q_{e}$ is the error quaternion, $q_{0}$ is the scalar part of the quaternion, and ω is the satellite angular velocity. The set of manoeuvres is defined both by varying the desired final attitude angles, including one-, two-, and three-axis manoeuvres, and scheduling the gains of the controller (with seven different gain variations).
- The s-measured quantities vary according to the type of sensor: s=15 acceleration time histories (i.e., five three-axis accelerometers are installed on the solar array), or s=6 voltages time histories (i.e., six piezoelectric patches are mounted on the panel, each of them producing one potential difference). In both cases, the collected data are arranged in a multidimensional array $X_{d}\in R^{s\times k\times q}$, with $k=N_{m}\cdot N_{d}$ and q number of time samples. Furthermore, a Gaussian noise equal to 2% of the measured values is applied to the time histories to simulate a realistic acquisition process and to improve the variability of data and, consequently, the robustness of the training. Specifically, since the process of identifying damage is approached as a classification problem, the output consists of individual entries that contain specific labels corresponding to attitude manoeuvres and damage configurations (refer to Table 3).

The multidimensional array $X_{d}\;$undergoes two consecutive pre-processing steps:

- Time sequence truncation: This phase is necessary to avoid including in the dataset those time samples that could reduce the performance the training process. Indeed, it was noticed that only the initial part of the measured signals contains a relevant dynamic content: they correspond to the excitation of the structural panels caused by the rigid attitude manoeuvre via the modal participation factors, when the torque control action and the induced elastic vibrations are the highest. On the other hand, including responses under a certain threshold (either acceleration or voltage) would have flattened the dataset, improving neither the training nor the classification accuracy.
- Data normalisation: This step is crucial to ensure the data are in the proper range of the dynamic variability in the learning space of the DL network. It was proven [25] that, for this type of application, normalisation with respect to the mean and standard deviation of samples offers better results than minimum–maximum processing.

**Figure 13:**
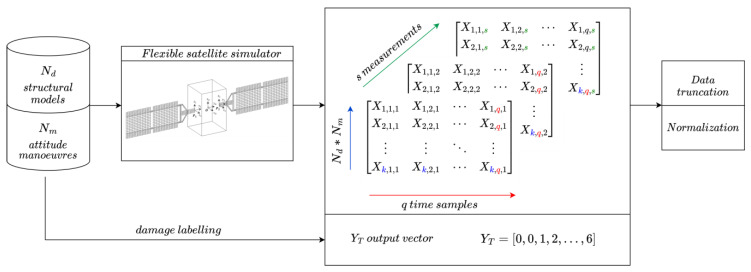
Training set generation and processing.

The final pre-processed dataset ${\bar{X}}_{d}$ is an array of size 15 × 1617 × 151 (for accelerometers) and of 6 × 1617 × 151 (for piezoelectric sensors), which is also the input to the DL architecture. Conversely, the output vector has dimensions of 1617 × 1, and assigns a classification label to each simulation (as indicated in Table 3). At the end of the procedure, the input–output pair is fed to the classification network for the training process, as depicted in Figure 13. The dataset ${\bar{X}}_{d}$ and output $Y_{Y}$ are then organised in the input/output pairs {*x, y*} mentioned in Section 4.1, when each time history is associated with one label, six different “damaged” classes, or an “undamaged” label. 

## 5. Experiments

The objective of this study is to examine the sensitivity and accuracy of the proposed architecture for identifying structural damage via sensors distributed across the monitored structure. To address this challenge, a DNN architecture specifically designed for multivariate time series classification was used, as described in Section 4. We carried out two different types of analyses: the first one to compare the performance of the two accelerometers and piezoelectric-based networks for damage isolation (i.e., to assess whether there is structural damage or not), and the second test to verify how the two systems behave in case of damage identification (involving detecting the exact location of the failure). 

### 5.1. Damage Isolation Results

**Figure 14:**
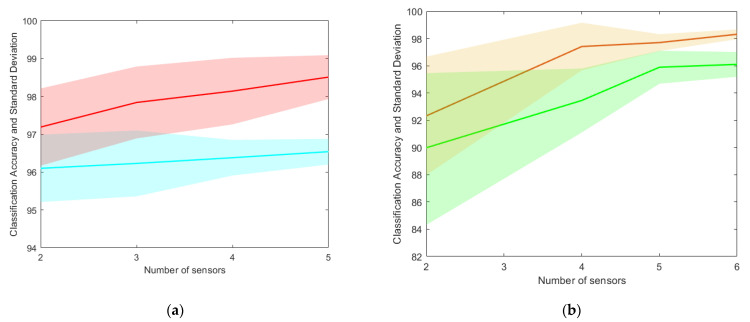
Classification accuracy variation with respect to number of sensors. The full lines indicate the mean of the accuracy for different sensor configurations, while the underlying opaque strips depict the accuracy standard deviation: (**a**) accelerometers—cyan line for the (0, 2) binary classification, red line for the (0, 5) binary classification; (**b**) piezoelectrics—green line for the (0, 2) binary classification, orange line for the (0, 5) binary classification.

Six binary classification problems were carried out, two for each damage scenario listed in Table 3 (one for the accelerometers and one for the piezoelectric sensors). The two classes are selected among the observation as follows: 231 time histories associated with the label “0” (i.e., undamaged panel) and 231 time series with a class label between “1” and “6” (i.e., damaged solar array). This analysis is performed to understand if the network can discern whether damage has occurred or not as opposed to the undamaged scenario. Moreover, such an assessment is also used to study how the classification accuracy changes by varying the number of sensors, to find a good configuration both in terms of accuracy and standard deviation. For the sake of brevity, only two representative cases—for both types of sensors—are illustrated in Figure 14. Finally, the proposed configuration includes five tri-axial accelerometers and six piezoelectric patches to maximise the accuracy and reduce its standard deviation. The results are illustrated in Table 5.

In addition, a further bi-classification problem is tested, and the results are listed in Table 6. Two classes of observations are fed to the network, with 231 time series having the class label “0” (undamaged system) and 924 times series having a “1” class label associated with all time sequences of the damage. It can be noticed that, also in this case—with an unbalanced dataset between the undamaged and damaged measurements—the classification architecture proves very effective. In this case, the network can confidently isolate the damage by discerning whether the signal in the input originated from an “undamaged” or a “damaged” structure. However, the system is not trained to identify the failure location.

### 5.2. Multi-Damage Identification Results

**Figure 15:**
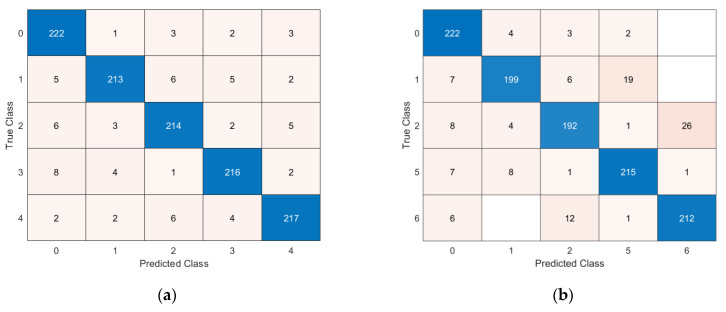
Confusion matrices for multi-damage classification with accelerometer sensors: (**a**) Case C, (**b**) Case D.

**Figure 16:**
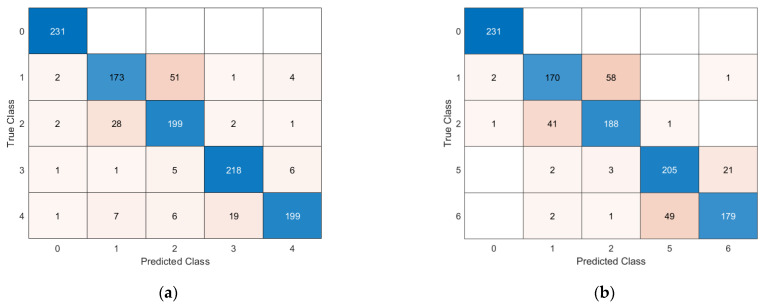
Confusion matrices for multi-damage classification with piezoelectric sensors: (**a**) Case C, (**b**) Case D.

In this paragraph, the results from a more challenging problem are presented. In detail, four cases of damage are considered in those areas associated with both the highest MSE (red areas in Figure 5) and lower MSE (green areas in Figure 5). Two cases were analysed: Case A includes only the damage in the red/orange areas with the objective of identifying cases of damage adjacent to each other, while Case B studies damage in both red and green areas, with the rationale of discerning failures in both the highest and lower MSE densities. Despite such damage inducing a very limited change in the system frequencies and modal shape (<< 1% relative difference), the DNN shows good classification accuracy. The classification accuracy is shown in Table 7, while the confusion matrices for the accelerometer and piezoelectric cases are illustrated in Figure 15 and Figure 16.

## 6. Discussion

The proposed network shows good classification accuracy in all analysed cases. In detail, while both solutions clearly show very good performance in the case of damage isolation (bi-class identification tasks in Table 5 and Table 6), the more challenging five-class identification problem (in Table 7) shows that the DL architecture works slightly better with data collected using accelerometers. A discussion of the findings is reported in Table 8.

It is also worth noting that a limited set of sensors proved sufficient to detect failures in the very first instants of the attitude manoeuvres (151 samples acquired with 20 Hz sampling frequency), thus offering a promising approach for the early detection of structural failures. 

## 7. Conclusions

This research aims to address the diagnostics of structural breaks due to space debris impact in complex systems, with a specific focus on large solar panels realised in a one-layer equivalent composite material. The study also investigated the sensitivity of the Structural Health Monitoring (SHM) framework and sensors to localise damage, by comparing the performance of the architecture when fed with two different sets of data, produced by piezoelectric and accelerometer sensors. To this end, a data-driven SHM approach utilising a state-of-the-art deep recurrent neural network named Bi-LSTM was implemented and tailored for damage classification. The results obtained from the study demonstrated a good effectiveness of the SHM system in accurately isolating breaks in the most critical areas of the panel (bi-classification problem), and in a more complex multi-class identification (five-class) problem. In detail, while being comparable in the case of the bi-classification tasks, accelerometers showed slightly better identification performance in the five-class problem. It should be remarked that the proposed damage identification end-to-end procedure was demonstrated to be very robust and effective, also found in previous authors’ work, when applied to data collected during satellite attitude manoeuvres.

Future work will consider the adoption of automatic validation and testing methods to effectively tune the complexity of the proposed network. Furthermore, the DL approach presented here is a numerical preparation phase propaedeutic to scale and transfer the DNN architecture on a testing rig reproducing satellite manoeuvres in a laboratory setting, to test the effectiveness of the approach in a relevant on-ground environment.

## Figures and Tables

**Table 1:** Spacecraft inertial properties.

Mass	Inertia			Size		
(kg)	(kg m²)			(m)		
	$J_{xx}$	$J_{yy}$	$J_{zz}$	X	Y	Z
300	125	125	50	1	1	2

**Table 2:** Aluminium Alloy Al 5052.

Property	Symbol	Value
Face layer thickness	$t_{f}$	1 mm
Honeycomb height	$h_{c}$	8 mm
Young’s modulus	$E_{f}$	70.3 GPa
Shear modulus	$G_{f}$	25.9 GPa
Poisson ratio	υ	0.33
Density	ρ	2680 kg/m³

**Table 3:** Damaged elements.

$ID_{d}$	Description	Damaged Element	Classification Label
-	Undamaged	-	0
1	Damage 1—red area (high MSE)	Elm 602	1
2	Damage 2—red area (high MSE)	Elm 437	2
3	Damage 3—orange area (medium MSE)	Elm 601	3
4	Damage 4—orange area (medium MSE)	Elm 436	4
5	Damage 5—green area (low MSE)	Elm 435	5
6	Damage 6—green area (low MSE)	Elm 600	6

**Table 4:** Network learnable parameters for the five-class classification problem.

Layer		Accelerometers	Piezoelectrics
Input Layer		-	-
Bi-LSTM 1	Input weights	[160×15]	[160×6]
	Recurrent weights	[160×20]	[160×20]
	Biases	[160×1]	[160×1]
Dropout		-	-
Bi-LSTM 2	Input weights	[80×40]	[80×40]
	Recurrent weights	[80×10]	[80×10]
	Biases	[80×1]	[80×1]
Fully connected	Weights	[5×20]	[5×20]
	Biases	[5×1]	[5×1]
Softmax		-	-
Classification		-	-
Total learnable parameters		9945	8505

**Table 5:** Classification results of the bi-class SHM problem: undamaged vs. one case of damage.

$\mathrm{Failure}\;(ID_{d})$	Class Labels	Sensors	Accuracy (%)
1	(0, 1)	A	97.02% ± 0.32%
		P	96.71% ± 0.60%
2	(0, 2)	A	96.54% ± 0.34%
		P	96.10% ± 0.91%
3	(0, 3)	A	96.14% ± 0.18%
		P	97.45% ± 0.98%
4	(0, 4)	A	96.54% ± 0.45%
		P	97.49% ± 0.79%
5	(0, 5)	A	98.41% ± 0.38%
		P	98.31% ± 0.36%
6	(0, 6)	A	98.10% ± 0.64%
		P	97.32% ± 0.58%

A: accelerometers, P: piezoelectrics.

**Table 6:** Classification results of the bi-class SHM problem: undamaged vs. all damage.

Cases	Class Labels	Sensors	Accuracy (%)
A	(0) vs (1, 2, 3, 4)	A	97.34% ± 0.33%
		P	95.58%± 0.96%
B	(0) vs (1, 2, 5, 6)	A	95.78% ± 0.75%
		P	95.45% ± 0.66%

A: accelerometers, P: piezoelectrics.

**Table 7:** Classification results of the five-class Structural Health Monitoring problem.

Cases	Class Labels	Sensors	Accuracy (%)
C	(0, 1, 2, 3, 4)	A	93.57% ± 0.49%
		P	88.62% ± 2.33%
D	(0, 1, 2, 5, 6)	A	90.02% ± 1.48%
		P	84.37% ± 3.58%

A: accelerometers, P: piezoelectrics.

**Table 8:** Results interpretation.

Cases	Accuracy (%)
Bi-classification (Undamaged vs. one case of damage)	Concerning the bi-classification problem results presented in Section 5.1 (see Table 5), a different trend in the accuracy behaviour can be noticed between the piezoelectric and accelerometer sensors, as illustrated in Figure 14. In detail, accelerometers generally exhibit a lower variation in both accuracy and standard deviation with the increase in the number of sensors. On the other hand, piezoelectrics show a noticeable dependency on the number of devices, both in terms of accuracy mean value and standard deviation, which decreases significantly with a few more installed patches. This behaviour seems to be explainable by considering the different nature of sensing measurements, the first based on nodal translational motion (which is mostly related to the points where the most relevant motion can be registered), and the other one on the local structural strain (as confirmed by most literature concerning piezoelectric sensors, generally disposed in a dense mesh on the inspected area [10,52], clearly also depending on the adopted SHM method). In general, both devices seem to have reached a “convergence” condition for the accuracy value; hence, the number of considered sensors was deemed optimised for the considered application.
Bi-classification (Undamaged vs. all damage)	The second bi-classification problem addressed in Section 5.1 (see Table 6) analyses the undamaged condition with respect to all other damage configurations. The DNN architecture proved to be able to classify the labels with accuracy higher than 95% in all analysed cases, even if the training dataset is unbalanced between the two classes (i.e., “undamaged” vs. “damaged”). This shows how the proposed approach can discriminate between a “healthy” signal and a damaged one, thus providing the in-orbit system with a reliable damage isolation functionality. Both sensor configurations exhibit comparable performance, with piezoelectrics showing a very similar accuracy for the classification of both high-MSE (labels “1” and “2”) and low-MSE damage (labels “5” and “6”). In this bi-classification case, the accelerometers slightly outperform the piezoelectrics in the case of high-density MSE elements (97% vs. 95%), proving to be more robust to a potentially unbalanced dataset for practical applications.
Multi-label classification	Regarding the multi-label classification problem (see Table 7), for both sensor categories, the class “0” is generally well classified, with piezoelectrics showing better performance for this specific task, with lower cases of false detection (first rows in the confusion matrices in Figure 15 and Figure 16) and limited false alarms (first columns in the confusion matrices in Figure 15 and Figure 16). Most false predictions happen when a single case of damage, i.e., its location, has to be assessed. It should be noticed, however, that failures $ID_{d}=1,3$ $\;\mathrm{and}\;ID_{d}=2,4$ are adjacent to each other (in an area of 5 × 10 cm^2^), which inherently complicates the classification problem in differentiating two very similar dynamic responses to damage. At the same time, failures $ID_{d}=5,6$ induce a lower effect on the system dynamics and are, therefore, less detectable than the others. Moreover, a different classification pattern can be observed between the accelerometer and the piezoelectric approach. The former shows more evenly spread misclassifications among classes “1” to “6”, with slightly worse performance—as expected—when introducing the lower MSE damage $ID_{d}=5,6$, particularly when classifying classes “1” and “5”, and “2” and “6”, which are aligned pairwise along the longitudinal axis *y* of the panel (see Figure 1). Piezoelectrics, instead, are more challenged by the classification of labels “1” and “2”, and, likewise, “5” and “6”, which are symmetrically placed with respect to the y axis and will likely measure a similar change in rotations at the extremities of the patches (as described in Section 2.2.1). Nevertheless, it should be remarked that the trained networks show good classification performance overall.

## Data Availability

Not applicable.
